# Multi-Elemental Analysis of Hair and Fingernails Using Energy-Dispersive X-ray Fluorescence (ED XRF) Method Supported by Inductively Coupled Plasma Mass Spectrometry (ICP MS)

**DOI:** 10.3390/molecules29040773

**Published:** 2024-02-07

**Authors:** Zofia Mierzyńska, Maria Niemirska, Kacper Zgonina, Tomasz Bieńkowski, Krzysztof Hryniów, Paweł Świder, Katarzyna Pawlak

**Affiliations:** 1 Masdiag, Żeromskiego 33, 01-882 Warsaw, Polandtomasz.bienkowski@masdiag.pl (T.B.); krzysztof.hryniow@pw.edu.pl (K.H.); pawel.swider@masdiag.pl (P.Ś.); 2 Faculty of Chemistry, Warsaw University of Technology, Noakowskiego 3, 00-664 Warsaw, Poland; 3 Institute of Control and Industrial Electronics, Faculty of Electrical Engineering, Warsaw University of Technology, Koszykowa 75, 00-662 Warsaw, Poland

**Keywords:** metal determination, hair and fingernails, pellet formation, solubilization of keratin, ED XRF, ICP MS

## Abstract

This work compared the multi-element analysis of human hair and nails using inductively coupled plasma mass spectrometry (ICP MS) with an easy, fast, cheap, non-destructive method using energy-dispersive x-ray fluorescence (ED XRF). The ICP MS-based method was more sensitive (over 30 elements could be quantified) and costly (requiring more time, samples, and chemicals). The EDX-based method required laboratory and certified reference materials made of hair for instrument calibration. It was less sensitive (16 elements could be quantified: S, Si, Ca, Br, Fe, Cu, Cr, Mg, Si, K, Mn, Ni, Zn, Se, Sr, Pb), but it allowed us to replace troublesome grinding with the dissolution of keratin-based material with an alkalic agent (tetramethylammonium hydroxide, TMAH) and the formation of stable-for-days pellets. This method is simple, enables automation, and, due to the modification of wells in the autosampler of the EDX system via the immersion of home-designed inserts, it requires smaller amounts of biological material and binder (down to 70 mg instead of 500 mg required by commercially available instrument) to perform analysis. It was concluded that the EDX-based method offers complementary selectivity and sensitivity to ICP MS with the possibility of sample reuse for further analysis.

## 1. Introduction

Humans are continuously exposed to toxic elements, such as Al, As, Cd, Hg, Ni, Pb, and Sn [1]. They do not play a biological role but can replace other essential elements, disturbing metabolic processes. Their presence in the body is undesirable, and permissible concentrations in the blood are measured by their negative impact on the body, organs, tissues, or cells. The beneficial effects of essential elements (such as Cu, Cr, Fe, K, Mg, Mn, Se, Si, and Zn) can be expected within a strictly defined concentration range, depending on their form and administration route. Their concentrations in the blood are higher than in the case of toxic elements. However, they should not disturb metal homeostasis. As a result, the acceptable concentrations of metals in human blood (as well as various tissues) depend on their biological activity and range from a few ng/mL to several hundreds of ug/mL [2].

Unlike acute poisoning, effects associated with chronic poisoning are much more common. Metals and metalloids can be inhaled (most often Al, Cd, Hg, and Pb in the form of fumes, dust, and cigarette smoke), administrated orally (e.g., As, Se, Sn, Pb in seafood, cereals, chocolate; Al, Co, Cr, Ni, Pb released from kitchen utensils or plumbing), and released from orthoses placed in the body (Co, Cr, Ni). The accumulation of metals in the body leads to changes in the functioning of organs and glands, such as the heart, brain, kidneys, bones, and liver [2]. This situation is of particular concern in developing countries that have increased their industrial activity, leading to intense exploitation of fossil resources and air pollution in industrial areas or urban agglomerations [3,4]. It is, therefore, not surprising that different studies have shown a positive association between metal levels in patients with diseases, such as Alzheimer’s disease, breast cancer, endocrine disorders, hypertension, rheumatoid arthritis, and childhood neurocognitive disorders [2,5,6]. For example, bioaccumulation of lead, cadmium, cobalt, chromium, and copper causes damage to the nervous system, kidneys, liver, and reproductive system [2,7,8]. Therefore, there is an increasing demand for cheap and easily accessible methods to control the level of exposure of workers in industry and diagnostic methods enabling the prevention and monitoring of the patient’s condition during therapy.

Monitoring the degree of environmental and food contamination by toxic elements is necessary. However, the analysis of air, soil, water, and food samples may not be conclusive in assessing the health risks caused by toxic elements [2]. For these reasons, monitoring of metals and metalloids in biological samples collected from humans has been identified as the optimal choice for assessing the impact of exposure to pollutants on human health [2,8]. The primary biological samples used for these analyses are urine, blood, hair, nails, and tissue collected during implant replacement [9,10]. Many metals in biological materials occur in trace amounts, requiring sensitive instrumental techniques. The most commonly used are atomic absorption spectroscopy (AAS) with atomization in a graphite furnace (in a few cases in a flame) [11,12,13,14], optical emission spectrometry [15,16], mass spectrometry [4,5,6,17,18] with plasma as a source of excitation or ionization of elements (ICP OES, ICP MS), and total reflection X-ray fluorescence spectrometry (TXRF) [19,20]. In addition to the high operating costs typical of these techniques, they require sample mineralization [5,6,16,21] or extraction combined with preconcentration [13,16,18,22]. Therefore, many methods to determine metal ions are being developed using electrochemical [23] or spectrophotometric techniques [24], the selectivity of which depends on metal ions’ leaching, enrichment, and chelating.

It should be noted that the content of an element in biological material does not have total diagnostic value if it is not presented concerning other elements, especially chemically similar ones. This way, the degree of advancement of metal homeostasis changes is described, which may better reflect the patient’s condition. For this reason, techniques such as ICP OES/MS that allow for the simultaneous determination of many elements offer high sensitivity, selectivity, and even atomic/isotopic specificity are most often used [6,21,25]. In the case of urine and blood, metals can be determined with ICP MS also in dissolved/diluted non-mineralized samples [26,27]. Despite offering the best sensitivity and selectivity, plasma techniques require sample destruction to transfer the components to the solution containing less stable forms of elements and multiple sample dilutions to determine macro-components [28].

Moreover, before each series of measurements, it is necessary to calibrate the instrument (using a matrix-matching approach [29]) and use internal standards (for different groups of elements) to compensate for fluctuations in the plasma power and changes in the interface zone (in the case of MS) [30,31]. Finally, the technique requires staff with high analytical expertise to identify interferences and apply different reaction or resolution modes, monitor isotope or emission ratios, and make mathematical corrections for MS and OES detection, respectively [32]. All this makes it worth looking for new methods for determining metals and other elements in samples of biological origin, for which plasma techniques will be complementary or used as a reference.

Another exciting solution is the application of energy- or wavelength-dispersive X-ray fluorescence spectrometry (WDX or EDX), which allows for the simultaneous determination of metals in a solid material [33]. This technique requires that the bulk material be homogeneous, representative of the sample, and dense enough to increase the efficiency of the emitted radiation relative to the reflected and scattered one [34]. These features are achieved by appropriately grinding and blending material with a suitable binder and pellet formation under high pressure (up to 20 bar) [19,35]. 

EDX was already proven to enable monitoring of Mg, Na, P, and S in pelleted ground hair with boric acid and comparing their relative—mathematically estimated—amounts [36]. It should be noted, however, that the grinding process carries serious challenges related to losses of the tested material and electrostatic interactions, leading to its granulation. Lowering the temperature and moistening the material by adding an organic solvent (acetone, methanol, isopropanol) increase the abrasion process’s efficiency but also increase the material losses [37]. Moreover, the authors stated that the amount of hair required for pellet formation is a bottleneck of XRF application in hair analysis [36]. Other researchers demonstrated for Zn that XRF calibration with keratin-rich reference material is required to obtain accurate amounts of elements in hair or nails [38]. Therefore, it creates the opportunity to create a cheap and ecological method for metal determination in easily collected samples of hair and nails. However, it requires a reduction in the amount of sample for analysis and appropriate calibration of the XRF instrument.

This work aimed to develop a method for determining elements using EDX in un-ground and non-mineralized hair and nails. The method was supposed to be simple and allow for automation for quick screening tests. Many problems related to the amount of material taken for analysis, its homogenization, and the preparation of a representative pellet were solved during its development. The proposed sample preparation and calibration method can be implemented for metal analysis using other ED/WD/TXRF systems. 

## 2. Results and Discussion

Since the pelletization of dissolved hair samples and their application toward calibration of the EDXRF have not yet been described in the literature, they will be discussed in detail. The method and most of the results obtained by ICP MS/MS used to verify the EDX-based method and characterize human hair samples used as laboratory reference materials (LRMs) are included in the Supporting Information (Tables S1–S5).

### 2.1. Parameters of the ED XRF Influencing the Sensitivity of the Method

Due to the lack of available certified pellets made of material of biological origin, at the initial phase, it was decided to use certified polyethylene pellets containing known amounts of Br, Cr, Cd, Hg, and Pb (Calibration Set for PE Analysis, VHG-ROHS-PE-SET1D, VHG Labs, Manchester, NH, USA). In this way, the impact of pellet homogeneity and thickness on the results’ quality was excluded.

Three parameters were detected that significantly affect the sensitivity and precision of the technique: the pressure, the collimator, and the measurement time. 

The air pressure in the detection chamber significantly influenced the sensitivity. However, this is not a controllable parameter. The vacuum level depends on the model of the instrument used. Initial work was carried out using the 7000 model, which enabled operation in low-vacuum conditions (below 50 Pa). When using the model 7000 device, the detection of light elements was only possible at contents above 200 ppm. In the detection chamber of model 8100, the pressure reaches 1–5 Pa. This improved the sensitivity up to 50-times for determining problematic light elements (e.g., Na, Mg, Ca, and K). 

**Figure 1:**
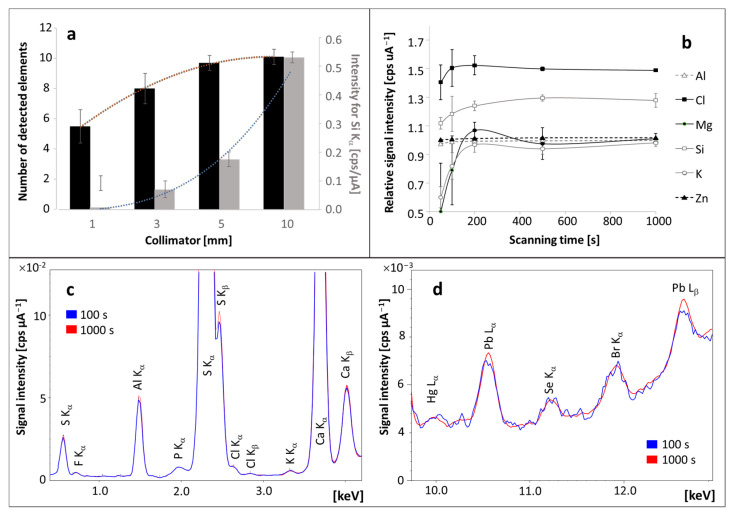
The effect of the radiation beam diameter on the height of the signals and number of detected elements in a pellet made of hair (**a**). The influence of scanning time on signal intensity and repeatability is presented in chart (**b**) and on spectra obtained for primary (**c**) and trace elements (**d**).

Increasing the diameter of the radiation beam (collimator) significantly increased the signal height for most of the detected elements and, thus, reduced the standard deviation and increased the signal-to-noise ratio (Figure 1a). As a result, more elements were detected after increasing the collimator diameter. It was decided to perform measurements for the largest permissible diameter of the radiation beam—10 mm.

The scanning time was another critical parameter affecting the signal-to-noise ratio (S/N). Analysis duration between 5 and 18 min offers the highest S/N (Figure 1b). It led to a more accurately defined spectrum, with reduced noise and improved repeatability of measurements (Figure 1c,d). As a result, the detection limit for As, K, and Mg and the lower quantitation limit for Cl, Cr, Hg, Mn, Na, Si, and Tl were lowered. On the other hand, the upper quantitation limit for Al, Br, Ca, Cu, Fe, Ni, Pb, S, Se, Sr, Y, and Zn was increased. 

It was also noted that in the case of a material with a complex matrix such as a biological one, the principle of obtaining better sensitivity for lower X-ray energy when determining light elements (smaller atomic radius allowing for more straightforward electron knockout) and higher energy for heavier elements is still fulfilled.

**Figure 2:**
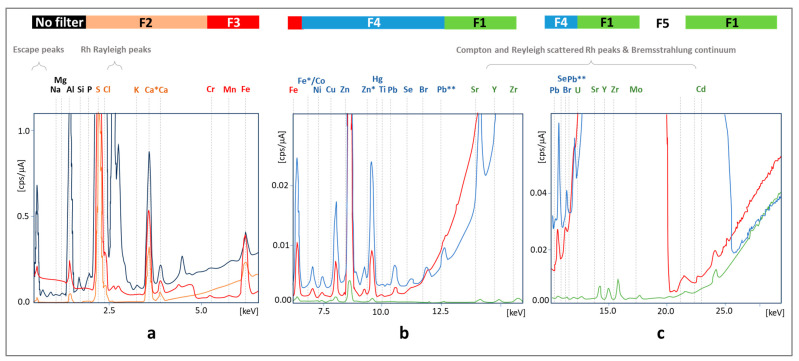
The influence of the type of filter on the attenuation of signals coming from the detection elements on the quality of the spectrum obtained for a hair sample for different X-ray energy ranges (**a**–**c**). The color of the spectral line corresponds to the background with the specified filter type above the spectra. Filter 5 was excluded from further studies. *, **—selected signals for elements represented by at least two peaks related to X-ray emitted when replenishing the K shell with electron—K_α_, K_β_, respectively.

Another problem is interference, which may be based on (1) radiation emitted by the X-ray source, leading to high background and disturbing signals in the spectrum; (2) X-ray radiation reflected by the sample atoms without loss of energy (Rayleigh/elastic scattering, Figure 2a), corresponding to the energies characteristic for the X-ray tube element (Rh); (3) scattered X-ray radiation without loss of energy due to the excitement of an inner-shell electron (Compton scattering); (4) other incidents related to Rayleigh and Compton scattering, leading to the presence of Bremsstrahlung continuum (high background, Figure 2b); (5) escaped peaks observed when the incident quantum energy exceeds the absorption edge energy of the detector element; and (6) fluorescence generated by secondary excitation of elements as a result of reabsorption of X-ray radiation by matrix elements. 

However, it is possible to use a filter between the radiation source and the sample to attenuate radiation in specific energy ranges (Figure 2). 

The best results were obtained for filters 1–4 made of aluminum, titanium, and copper, with different relative thicknesses of the layers of these metals. For low energies in the case of the determination of light elements (Ca, K, S), the best results were obtained for filter F2; for medium energies applied to determine Cr, Fe, and Mn, filter F3 and filter F4 made it possible to reduce the background to the greatest extent without signal loss in the case of Br and Pb atoms (Figure 2b); for the highest energies, the most elements could be detected after suppressing the radiation emitted by rhodium with filter F1. The average intensity of the generator was 100 A due to the need to limit the influence of the organic components of the matrix on the signal height (in the case of metal alloys, the intensity was 3–10-times lower). The collected optimal operating parameters of the EDX instrument used during the analysis of the tested biological samples are presented in Section 3.7.

### 2.2. Modification of Autosampler’s Wells

**Figure 3:**
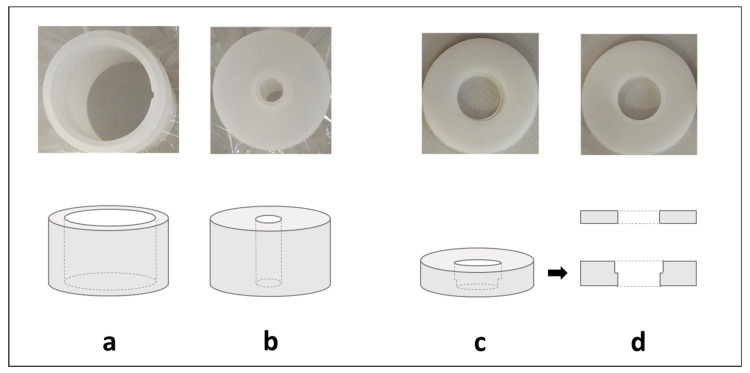
Simplified schemes and pictures of commercially available vials for typical samples (**a**) and low amounts of samples (**b**) with mylar or polypropylene film to secure the sample. Simplified schemes and pictures of the manufactured sample-well reducer used to place 13 mm pellets in the EDX detector chamber (**c**) and cover (**d**) to stabilize the position of the pellet immersed in the well-reducer (**c**).

In the case of material of biological origin, the sample amount available for analysis is limited. Hair and nails are a perfect example because a large volume of material has a low mass. To prepare a standard-sized EDX pellet (30 mm diameter × 5 mm thick), 0.5 g of hair or nails is needed. Meanwhile, an average of 0.1–0.2 g of material from one nail’s cut can be expected. The apparatus manufacturer offers vessels that reduce the amount of the necessary sample for measurement. However, they force a change in the diameter of the collimator, which reduces the sensitivity of the method (Figure 3a). It was decided to make an insert, reducing the diameter of the sample well in the EDX detection chamber, allowing for the placement of pellets with a diameter of 13 mm (Figure 3b). The second part of the reducer (cover) was used to block the position of the pellet, which was of particular concern when the appropriate vacuum was established. 

Sample well reducers were initially made of steel, but it turned out that the elements present in steel (Fe, Cu, Ni) are visible in the spectrum, regardless of the width of the X-ray beam (collimator). Once the polyoxymethylene (MOP) insert was made, a 10 mm diameter beam could be used, and no signals from the insert were observed. The minimum thickness of the pellet was also checked to ensure adequate X-ray absorption efficiency and to obtain the highest possible fluorescence. It turned out that the fluorescence increased with the thickness of the pellet up to 3 mm and then reached a plateau (Figure S1). It should be noted that it was established for hair, and the value may change depending on the sample type, binder, and determined elements.

This way, the amount of sample necessary to obtain a 13 mm pellet was reduced to 0.2 g when no binder was used. MOP-made reducers were placed in all 12 wells of autosamplers, and their cohesion was checked by establishing the precision and trueness by measuring the metals in certified polyethylene (PE) powder, pelleted and placed in all 12 wells with an inserted well reducer (RSD for Br, Cd, Cr, Hg, Pb was lower than 2%).

### 2.3. Formation of Pellets for Calibration of EDX

**Figure 4:**
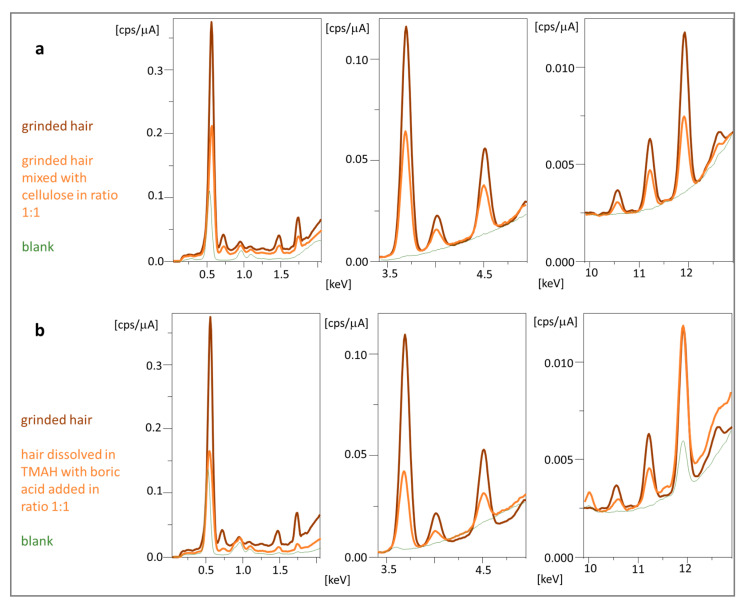
EDX spectra obtained for pellets from ground hair compared to (**a**) ground hair mixed with cellulose used as a binder and (**b**) hair dissolved in TMAH mixed with boric acid as a binder.

Pellets made from ground hair without adding a binder were quite unstable. After 24 h, they began to crumble due to moisture absorption from the air. The addition of a binder in the form of cellulose solved this problem. Wax as a binder melted during pelleting, which increased the variance of the method. In turn, using boric acid increases the friability of a tablet. Cellulose can be used in ratios of 1:1 and 2:1 to the tested material (added in mortar and mixed accurately), which reduces the required amount of tested material to 50 mg. The stable cellulose-hair pellets for up to two weeks provided good precision for 10 tested elements (RSD 3.97% for n = 12), but they were stored in vacuum bags or a desiccator. The spectrum of the hair pellet obtained with the cellulose binder is shown in Figure 4a. In addition, the spectrum derived from cellulose is shown, based on which it can be concluded that cellulose as a binder is not a source of interference and can be used to determine metals in biological material.

Hair and nails are a significant challenge when grinding (electrostatic effects are reduced by adding a small volume of polar organic solvent; need to use grinders with a small chamber to reduce waste, avoiding contamination of the material by metals from elements made of steel). Using agate instead of steel significantly extends the time of the grinding process (from 3 min to 1 h), which leads to an increase in the temperature of the material. Therefore, it is necessary to cool the grinding bowl properly. It can be achieved with liquid nitrogen (incompatible with agate) or by discontinuous grinding (for example, 10 min of grinding interrupted with 5 min pauses). Still, it can be problematic when a higher number of samples must be prepared. Therefore, a second method of preparing pellets from a material that is not ground but is dissolved has been developed so that after evaporation of the solvent, it reaches an appropriate/expected homogeneity.

Samples were dissolved using a 25% TMAH (*m*/*v*) solution in methanol. Methanol shortens the sample drying time before pelletization, making sample preconcentration more efficient. The obtained biomass was viscous and made it possible to obtain more durable pellets with boric acid as a binder (RSD 2.97% for n = 12 measurements carried out within 10 days). As a result, three calibration methods were obtained: using ground (G), dissolved hair, and gelatin matrix spiked with metal ion standard solutions (TH, TM). In each case, the slope coefficients of standard curves for the tested elements differed significantly (usually by 3–5-fold, Table S5). The difference is caused by matrix components (cellulose vs boric acid, gelatin), changes in metal speciation, structure of protein, and pellet density. It means that the matrix influence on signal intensity is significant, and the instrument calibration method should consider it. Despite a similar background, pellets based on gelatin offered higher sensitivity (slope coefficients), and adding boric acid instead of cellulose enabled the detection and determination of the most significant number of elements. 

It should be noted that sample preparation via dissolution with TMAH is more manageable to automate and increase throughput than grinding. Moreover, TMAH-dissolved samples offer better homogeneity and stability than ground hair or nails. Also, detection limits established for the blank were lower for most elements when a procedure based on TMAH was used. 

The proposed TMAH-based method for sample preparation does not require hair grinding. It can also be used for metal determination by ICP-MS. This way, microwave-assisted mineralization, which requires high amounts (5–7 mL per sample) of concentrated nitric acid and hydrogen peroxide, can be avoided. Moreover, hair pellets can be redissolved or mineralized after analysis using EDX for further ICP MS-based determinations. However, the background will be increased by boric acid and other chemicals added during the pellet preparation. For example, the presence of Pb in the blank sample (signal L_β_ observed at 12.6 keV) is subtracted mathematically.

### 2.4. Validation of EDX Method for Determination of Selected Elements in Hair

It was decided that metal determinations would be performed in hair dissolved with TMAH, increasing the method’s throughput and significantly reducing its cost. For EDX calibration, pellets were prepared by mixing hair in various proportions containing different and known metal contents (two types of CRMs and two LRMs with determined metal amounts by ICP MS). 

The metal content in the CRM hair samples is higher than usually detected in randomly collected human samples; therefore, to determine the LOD of the EDX-based method and increase the linear range of the calibration curve, mixed hair samples (discards from the hairdressing salon) were subjected to demetallization by sequential washing of hair in heated (80 °C) solutions of 10% nitric acid, 3.6% hydrochloric acid, and 10 mM ammonium acetate solution with 50 mM EDTA and 50 mM thiourea. Each step lasted 60 min and was followed by hair rinsing with MiliQ water. Chemically leached hair contained reduced amounts of metal ions, detectable by ICP MS but not by EDX. Such a hair sample (LRM1) was used to determine the LOD (blank) and to mix with metal-rich hair for weight-controlled dilution (as LRM 2). 

The second batch of collected mixed hair was incubated in a solution of metal ions to adsorb them on the hair surface. A sample enriched with metal ions and amounts established by ICP MS (LRM2 was used to establish the upper quantitation limit. It should be noted that the linear response range does not depend on the detector’s dynamic range but on keratin’s ability to adsorb metal ions. 

The hair used for EDX calibration had to be ground to mix efficiently with commercially prepared certified materials. The homogeneity of prepared LRMs is presented in Figure S1. Calibration pellets were prepared from TMAH-dissolved reference materials according to protocols described in detail in Section 3.4 and Section 3.5.

The obtained calibration curves with statistical descriptions are presented in Table 1. EDX offers poorer sensitivity than ICP MS for the determination of elements, such as Ag, Ca, Cd, Co, K, Mg, Mn, Na, V, and Zn, but better for Al and Si because samples were prepared in one vessel using a highly effective 25% methanolic tetramethylammonium hydroxide alkalic solvent (TMAH), reducing the risk of contamination [39]. Sulfur and selenium are also more precisely determined using EDX because the elements are interfered with in ICP MS by highly abundant polyatomic ions of oxygen ^32,33,34^O_2_^+^_,_ and argon ^74,76,78,80^Ar_2_^+^, respectively. Their efficient decomposition using collision or reaction gas comes at the cost of lower ion transmission to the electron multiplier.

Instrument stability was tested for two months using a reference metal alloy (stable in time regardless of storage conditions). Metals were determined (n = 15) using a method established for hair and nails. Relative standard deviation was below 2% for most elements, except chromium (4.3%), sulfur (6.8%), magnesium (6.1%), and lead (4.1%). Higher variability for the sulfur and magnesium amounts was related to their quantity below LOQ. It can be concluded that the EDX instrument can operate stably and return to the expected conditions after a week of non-use without recalibrating the instrument. Meanwhile, the ICP MS instrument was calibrated every day before sample analysis due to plasma fluctuation and changes in the dimensions of the interface orifice exposed to corrosive acids and salt solutions.

Four pellets were made to check the repeatability for manufacturing the pellets for one type of hair, each on a different day. Measurements were made three times for each pellet to determine the coefficient of variation for the sample preparation process vs. instrumental precision. The obtained variation coefficients (Table 2) are higher for the sample preparation step than for repeated measurements obtained for one pellet. The exception is the coefficient of variation for silver, which is below LOQ.

The method’s accuracy was positively verified for 13 elements in two CRMs (Table 3 and Figure S3), even if their amount was lower than 1 ppm, and these elements were selected for further tests. For four elements (Ag, Co, Cd, Mo, V), the trueness of the method was confirmed only for one CRM because the amount of elements was lower than 0.1 ppm (below the LOQ of the method). Light metals with a small number of orbitals, such as Na and K, proved to be troublesome to determine due to the inconsiderable energy dispersion. Accurate determination of vanadium was impossible in both CRMs due to interference from Cr.

### 2.5. Application of EDX Method for Determination of Selected Elements in Nails

**Figure 5:**
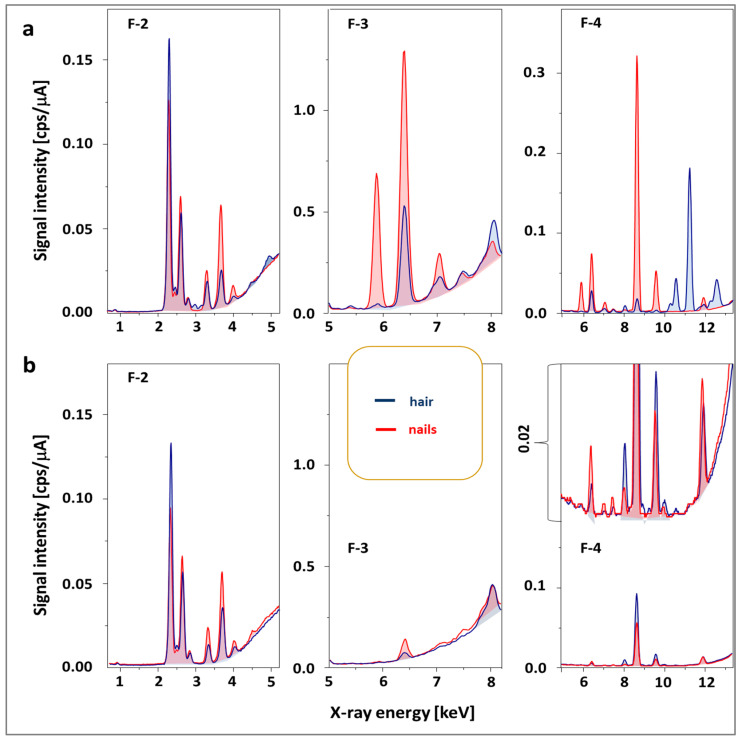
ED-XRF spectra obtained for hair and nails containing the highest (**a**) and the lowest (**b**) amounts of metals in examined samples.

Given that keratin is the basic microfiber from which hair, nails, hooves, and bird beaks are made, it was decided to check whether the developed method for determining elements in hair could be applied to human nails. Nail pieces were collected and ground (with the addition of acetone as an anti-granulation agent) to obtain a material with a particle size lower than 20 μm (verified using optical microscopy) and divided into two representative portions. Samples prepared the same way as hairs were subjected to metal determination by ICP MS/MS and EDX. A comparison of the ED XRF spectrum obtained for the hair with that obtained for the nails confirms the assumption that the composition of the sample matrix is spectrally significantly similar (Figure 5). However, nails have a greater ability to accumulate selected elements than hair. These are as follows: Ca (K_α_ 3.7 keV), Mn (K_α_ 5.9 keV), Fe (K_α_ 6.4 keV, K_β_ 7.1 keV), Ni (K_β_ 8.3 keV), Zn (K_β_ 9.6 keV). The sample was diluted using a binder, and the result was recalculated according to the nail/binder ratio. It is worth noting that spectral background and signal height with increased additions of boric acid changed in the same way as for hair samples, leading to the conclusion that the same calibration can be applied for both hair and nail samples.

A comparison of the results obtained by ICP MS/MS and ED XRF methods shows that in both methods, in the case of 15 elements, the determined amounts agree within the range of the expanded uncertainty of the method (k = 2, Table 4). However, it should be noted that for chromium and nickel determination by ICP MS/MS, it was necessary to use platinized cones instead of the standard nickel-plated ones to avoid a positive error.

Metal amounts in nails are similar to those obtained for hair used for EDX calibration and, in this study, were mostly within the linear response range of the method established using certified hair reference materials. If the amounts of metals exceeded the upper limit of the linear range of the method, the pellet was ground in a mortar with the addition of boric acid and pressed again prior to re-analysis. The determined metal content was recalculated, considering the degree of dilution. 

The trueness of the EDX method was confirmed (by comparing the results obtained by EDX and ICP-MS/MS) in the case of 15 elements. However, it should be noted that the bromine, chlorine, mercury, and silicon determination was performed using ICP MS/MS only in a pooled sample (combined material of various biological origins). As the amount of nail material is limited, acid mineralization was selected because it allowed for the determination of more elements than the TMAH-based method. Samples in the form of solutions had to be analyzed within 6 h. After 18 h, the recovery of many elements (Ag, Al, Ba, Cd, Cl, Cu, Fe, Zn) decreased by up to 60%, and the precipitate was observed at the bottom of the volumetric flask. 

The EDX method was more accurate than ICP MS/MS for determining bromine, silicon, sulfur, chlorine, mercury, and selenium. This was due to the different chemical nature of the elements, which required a different method of digestion or stabilization of the elements in the solution. The ICP MS/MS method was more accurate for determining cadmium, cobalt, manganese, molybdenum, and silver.

Moreover, it should be noted that MQ-water (resistance 18 MΩ) obtained by the Milipore system used in our laboratory was sometimes contaminated with Sr. Two additional purification systems were tested, and one ensured adequate purity of the water used as a dilution medium. This is substantial as the sample needed to be diluted by factors of 500, 1000, and 10,000 to determine different elements in the mineralized sample. Such problems were avoided in the EDX-based method, as the sample was usually diluted only two- to four-times with boric acid before pellet formation.

### 2.6. Applicability of the EDX Method Considering Biological and Environmental Diversity of Samples

**Figure 6:**
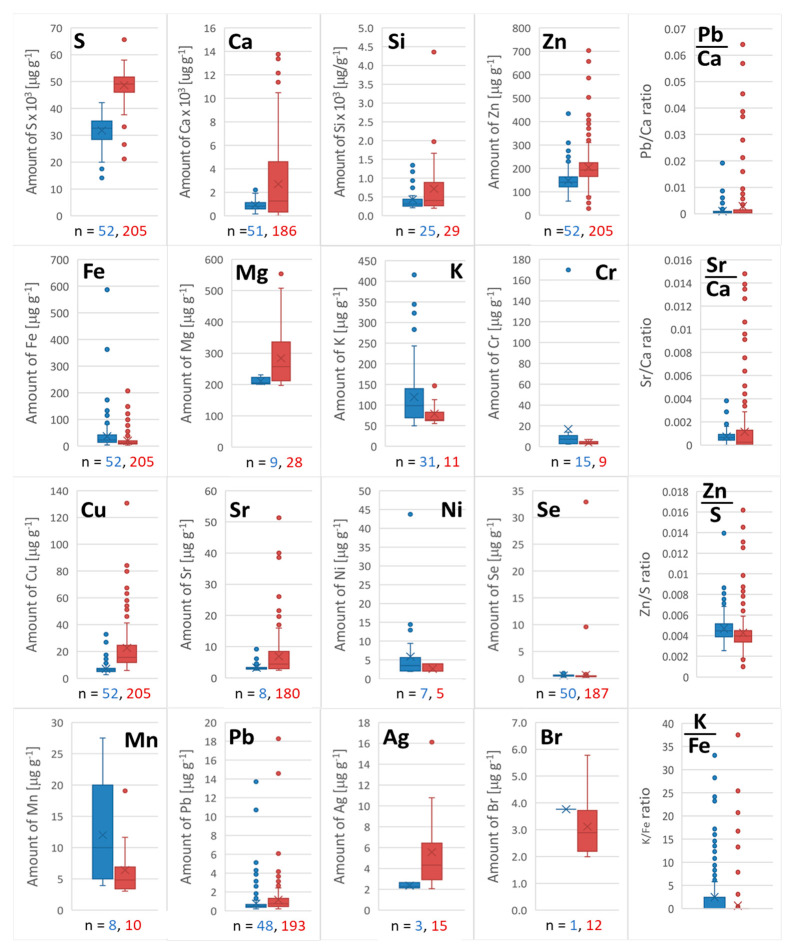
Box charts illustrating the distribution of obtained results for selected elements determined in nails (blue) and hair (red) and ratios for selected elements.

Discarded hair and nail samples were collected from beauty salons in Warsaw. Hair of different colors was fractionated and classified as the sample obtained from one individual. The nails collected at one station were combined and considered as one sample. The obtained results for 208 samples of hair and 52 of nails are presented in the form of box charts to show data distribution as an interquartile range, median, and statistical dispersion and indicated outliers (Figure 6). The number n in the chart is the number of the results above LOQ of the EDX-based method. 

It can be easily noticed in Figure 6 that for elements, such as Ag, Br, Ca, Cu, Mg, Mn, S, Si, and Sr, the interquartile range was the widest. In the case of Ag, Br, and Mn, the number of the results was very low, and statistical dispersion was very high. This is the effect of a low signal-to-noise ratio, typical for amounts close to the lower limit of quantitation. Distribution of amounts for Ca, Cu, Mg, S, Si, Sr, Fe, and Zn may result from various commonly used care treatments, such as cleansing, conditioning, coloring, and styling [40]. It can influence the hair fiber’s condition and increase its ability to adsorb metal ions. All these elements, except strontium, are essential for humans, which can cause a risk to health in the case of prolonged exposure, resulting in the replacement of calcium ions. 

The ratio of Ca/Sr and Ca/Pb amounts in the hair and nail samples should be above 700 and 1100 in hair and nails, respectively [41]. Meanwhile, for three samples of hair containing outlier amounts of Sr and Pb, the ratio was below 60 and 33, respectively. Unusual amounts of strontium and lead in hair compared to other hair of Warsaw residents exposed to similar water and air quality may indicate long-term exposure to toxic elements. A similar effect was observed in two mixed nail samples as the excess of Ca to Pb was below 100.

In the case of nail samples, the most diverse results were obtained only for K and Mn. Potassium can be monitored additionally as a ratio of K/Fe. The dominating ratios were up to 2.5, but a ratio above 20 was obtained for four samples. Such a significant difference may be an effect of metal homeostasis disturbance, which may result from incorrect supplementation, a side effect of medications, or severe iron deficiency [42]. Detected aberration should, of course, be verified by determining the metal content in the blood to exclude contamination or interference.

It should be noted that the ranges for metal amounts obtained by the EDX method were consistent with the literature data [43,44], including lead and strontium amounts comparable to essential selenium.

High amounts of Ag detected in 15 hair samples were surprising but probable. The application of cosmetic products containing silver is rapidly increasing in EU countries due to marketing practices and health claims. Therefore, the presence of silver in hair and nail samples is justified. However, due to the proven toxicity of silver in the form of ions, colloids, and nanoparticles, its presence should be carefully monitored [45].

## 3. Materials and Methods

### 3.1. Chemicals

Acetone and methanol of HPLC grade, nitric acid, hydrochloric acid, and 25% (*v*/*v*) hydrogen peroxide of purity for trace analysis were purchased from VWR (Darmstadt, Germany) and Sigma-Aldrich (Saint-Quentin-Fallavier, France). A 25% (*m*/*v*) tetramethylammonium hydroxide (TMAH) solution in methanol and a second in water were purchased from Alfa Aesar (Kandel, Germany) and JKchemical (Pforzheim, Germany), respectively. Boric acid of analytical purity (SO-press001) was purchased from PD Instruments (Kleve, Germany). Environmental Calibration Standard (5183–4688) containing metal ions at two concentration levels was purchased from Agilent Technologies (Stara Zagora, Bulgaria). Three certified reference materials (CRMs) prepared from human hair (NCS DC73347a and NCS ZC 8100 2b, CISRI, Beijing, China, and ERM-DB001, ERM, Geel, Belgium) were used toward calibration of ED-XRF and methods’ validation. Milli-Q Integral Elix system (Merck Milipore, Darmstadt, Germany) was used to prepare ultrapure water (18.2 MΩ/cm).

### 3.2. Instrumentation

Hair and fingernails were homogenized using a planetary ball mill (Fritsch, Oberstein, Germany) with a single grinding station made of agate to avoid contamination with metals. Thus, 13 mm pellets were obtained by compression of powdered hair and nails using up to 15 tons of force with hydraulic press (PIKE technologies, Madison, WI, USA). Metals in pellets were determined using an energy-dispersive X-ray fluorescence spectrometer (ED-XRF, Model EDX-8100, Shimadzu, Kyoto, Japan) equipped with a vacuum pump. Special plastic reduction inserts (matching the wells of the autosampler) were made of polyoxymethylene (MOP) to place and lock smaller 13 mm pellets in the center of the autosampler’s well.

Hair and nail samples were mineralized using SpeedWave MWS-3 microwave (Berghof, Eningen, Germany). Metals mineralized and dissolved in TMAH hair and nails were determined using inductively coupled plasma mass spectrometry (ICP MS/MS, Model 8900, Agilent Technologies, Tokyo, Japan) equipped with SPS4 autosampler and MicroMist nebulizer (0.4 mL min^−1^) with ratchet gas fitting.

### 3.3. Hair/Nail Washing and Homogenization

Hair and nail discards were collected from several beauty salons (Warsaw, Poland). Hair was then fractionated according to color and texture, originating from a single source, while nails were collected together as one sample at each workstation. 

Samples were placed in clean 30 mL (hair) and 10 mL (nails) glass containers. The material was subjected to sequential washing with acetone, deionized water at 40 °C, and acetone enhanced with shaking at 320 rpm for 20 min (water bath WN 22, Memmert GmbH, Buechenbach, Germany) [46]. Once the washing was complete, the samples were filtered and left to dry at room temperature on a filter in a Petri dish. 

Samples were pre-cut with ceramic scissors and placed in the bowl of the mill. To prevent electrostatic interactions and granulation of the material, acetone was added to the material at a ratio of 1 µL per 3 mg for hair and 3 µL per 1 mg for nails. The samples were ground for more than two hours in cycles (3 × 30 min grinding, 2 × 30 min cooling). The obtained powder was filtered and left to dry at room temperature on a filter in a Petri dish; next, it was inspected with a microscope for homogeneity and particle size (Figure S1).

### 3.4. Preparation of Pellets from Hair and Nail 

**Figure 7:**
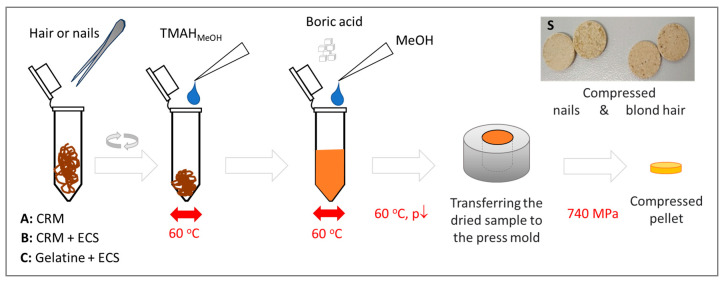
The scheme of preparing hair and nails (A) for measurement using ED-XRF and reference pellets (B,C) for calibration. CRM—certified hair reference materials, ECS—environmental calibration solution containing metal ions, S—photographs of pellets prepared for measurements.

100 mg of test material (hair/nails) was weighed into an Eppendorf tube, and 250 µL of TMAH solution was added. Solubilization was enhanced by vortexing the sample at 4200 rpm for 1 min at 60 °C. After 2 h, samples were centrifuged at 10,000 rpm for 6 min and incubated at 60 °C for 1 h. Next, 50 µL of methanol and 250 mg of boric acid were added to the resulting suspension and mixed thoroughly at 4200 rpm for 1 min (Figure 7).

The mixed sample was dried in a desiccator under reduced pressure (680 mmHg) for 1 h and placed on a heating plate for 2 h at 60 °C. The dried sample was transferred to a hydraulic press, and the pellet was formed within 2 min at a pressure of 10 tons. Prepared samples were analyzed within 24 h. Otherwise, they were stored in vacuum bags or containers to protect against moisture. Samples were prepared in a set of 12 (number of wells in the autosampler).

The chamber and piston of the hydraulic press were washed with acetonitrile and methanol using a dust-free wipe to prevent sample carry-over.

### 3.5. Calibration of EDX

Pellets for calibration of EDX were prepared following the procedure described in Section 3.4 but with two types of modifications: (A) different amounts of CRM powder were weighted (Table 5) to obtain different dilution factors against a fixed amount of binder, and (B) different amounts of metal ions were added using environmental calibration solution (ECS, Table 1 and Table 5) to obtain different levels of dissolved CRM. Suspensions were prepared in Eppendorf tubes, shaken, dried, and pressed, as previously described.

Amounts of metals in calibration pellets (C_cal_) were calculated according to the following equations:

(1) $$A:\;\;\;\;\;\;\;C_{cal}=\frac{x}{100}\times C_{CRM}\left[\frac{µg}{g}\right],$$

where *x* is the weight of CRM according to Table 1 (mg), 100 serves as the correction factor representing fixed weight for investigated samples (mg), and *C_CRM_* as element content in CRM (µg g^−1^).

(2) $$B:\;\;\;\;\;\;\;\;\;\;C_{cal}=\frac{0.05\times C_{CRM}+C_{st}\times \frac{V_{st}}{1000}}{0.1}\left[\frac{\mu g}{g}\right],\;$$

where 0.05 corresponds to the fixed weight of the CRM powder (g), *C_CRM_* represents element content in CRM (µg g^−1^), *C_st_* and *V_st_* represent element concentration in the standard solution (ECS) (µg mL^−1^) added in appropriate volume to CRM (µL), 1000 is a conversion factor for volume unit from µL to mL, and 0.1 serves as correction factor representing fixed weight for investigated samples (g).

### 3.6. Mineralization and Solubilization of Hair and Nails toward Metal Determination by ICP MS/MS

3.5 mL of concentrated nitric acid was added to about 0.15 g of hair or nails in a PTFE vessel. After 1 h, 1.5 mL of hydrogen peroxide was added. After waiting a minimum 2 h, microwave-assisted mineralization was performed using a Speed Wave Microwave (Berghof, Eningen, Germany). The program (Table S1) was established by comparing the obtained metal recovery (as high as possible) and method variance (as low as possible) with certified amounts of elements in reference hair material. After mineralization, the sample was quantitatively transferred to a 20 mL volumetric flask and analyzed on the same day because the stability tests of the samples showed that for some elements, the signals decreased to 20% after 18 h and to 45% after 48 h.

The second sample preparation method was the solubilization of hair and nails. A sample of 0.2 g of hair or 0.1 g of nails was placed in a polypropylene falcon tube. The appropriate amount (5 and 2.5 mL, respectively) of concentrated TMAH solution (25%, *v*/*v* in water) was added, and a falcon tube was sealed. After ~12 h of incubation at 30 °C, the other reagents were added to the samples (thiourea, triton, and ethylenediaminetetraacetic acid) in the amounts shown in Table S2 and mixed thoroughly. Next, samples were quantitatively transferred to 20 mL volumetric flasks and analyzed on the same day.

### 3.7. EDX and ICP MS/MS Detection Conditions

Optimal conditions to determine elements by EDX-8100 were established using pellets prepared from human-hair-certified reference material NCS DC73347a to detect the highest number of elements and obtain the highest S/N ratio. Optimal detection parameters for ICP MS/MS (RF power, nebulization parameters) were established using a tune mixture containing 1 ng mL^−1^ metal ions (Li, Co, Y, Ce, Tl) in 5% nitric acid. Next, an environmental standard solution containing trace metals at a concentration of 5 ng mL^−1^ was used to establish optimal collision/reaction cell conditions. Final integration times and isotopes were established after analysis of human hair and nail samples according to their amount in the samples. Optimal parameters for both techniques are presented in Table 6.

### 3.8. Calibration of ICP MS/MS

ICP-MS was calibrated using solutions containing calibration standard diluted in a 2% (*v*/*v*) nitric acid solution (for samples after mineralization) and 1% (*v*/*v*) TMAH solution containing 0.25% EDTA and 0.025% Thiourea and Triton (for dissolved samples).

The following standard solutions were used to prepare the calibration for (A) selected non-metals (S at concentration 10 μg·mL^−1^), P, I, Br (at concentration 0.1 μg·mL^−1^) prepared in water and for (B) metals/metaloids Fe, K, Ca, Na, Mg at a concentration of 10 μg mL^−1^ and Ag, Al, As, Be, Cd, Co, C, Cu, Mn, Mo, Ni, Pb, Sb, Se Tl, V, Zn, Th, U, Sn, Hg, Sr at a concentration of 0.1 μg·mL^−1^ in 2% HNO_3_ aqueous solution. Precision and recovery were compensated by a signal obtained for internal standards (Ge, Y, Rh), with a concentration of 10 ng mL^−1^ in each solution.

The linear response range for micronutrients was 0–80 ng mL^−1^, while for macronutrients, it was 100–8000 ng mL^−1^. The sequential dilution of the sample enlarged the applicability of the method. A statistical description of calibration graphs is presented in Tables S3 and S4.

### 3.9. Data Registration and Analysis

MassHunter 11.0 Software registered data obtained by ICP MS/MS, including calibration curves and their statistical analysis. Data obtained by ED XRF were collected using Shimadzu PCEDX-Pro Ver.2.05 software. The part of software applied to create reports containing quantitative analysis data was modified to transfer data without mathematical rounding of numbers (it led to the loss of significant changes in signals for elements present at trace levels as software was prepared for an old 7000 model able to determine only major components) and collect them combined in a spreadsheet (see Section S3 of Supplementary Material). Obtained data for screening tests were analyzed using STATISTICA 13.1 and Excel (Microsoft 365 Office) software.

## 4. Conclusions

Energy-dispersive X-ray fluorescence (ED XRF) can be applied to determine essential and toxic elements in human hair and nails. An entire spectrum is acquired practically simultaneously so that 16 elements (S, Si, Ca, Br, Fe, Cu, Cr, Mg, Si, K, Mn, Ni, Zn, Se, Sr, Pb) can be determined within 20 min, including the use of filters necessary for the analysis of trace elements.

Laboratory or certified reference materials (LRMs and CRMs) are necessary for instrument calibration to determine elements accurately. However, they can be diluted with binders to obtain durable pellets.

An alternative method for the sample preparation of hair and nails without using a mill was developed. Samples were dissolved by a highly effective alkalic medium (tetramethylammonium hydroxide, TMAH), preconcentrated, and mixed with a boric acid binder in one small tube. Such a “one pot” approach reduces loss of the hair/nail, diminishes the risk of contamination, and enables automation. Mixed hair samples were ground only when preparing laboratory reference materials to enable the determination of elements by ICP MS after mineralization. Obtaining LRMs was time-consuming (2–3 h interval grinding, 3.5 h sample conditioning with acids, 1 h mineralization and cooling), but the most critical step was grinding. The grinder allows for the preparation of only individual samples and is responsible for the loss of material adsorbed on its abrasive parts. Dissolving samples with TMAH is, therefore, a better option than grinding when preparing small amounts of samples for pellet production.

The developed method of producing smaller pellets and modifying the wells in the autosampler met the requirements of the limited sample size in the case of nail analysis. 

The obtained pellets are more stable than solutions prepared for analysis using ICP MS. They enable repeating the analyses, dissolving the pellet, and analyzing its contents using ICP MS or ICP MS/MS.

EDX is not as sensitive as ICP MS. However, it allows for the determination of many elements that cause problems to ICP MS due to the high ionization energy of elements (Br, Cl, S, Se, and Si), numerous interferences (Al, Cr, Pb, S, Se, Si), or the need to repeatedly dilute the sample, increasing the risk of sample contamination (for example, with Sr).

To sum up, the EDX method provides complementary results to those obtained using ICP MS in the tested materials of biological origin.

## Figures and Tables

**Table 1:** Statistical characteristics of calibration graphs obtained for selected elements by EDX in pellets obtained for hair dissolved by TMAH and containing boric acid as a binder.

El.	y = ax + b	r2	Linear Range [ug/g]	n	SD_a_	SD_b_	LOD	LOQ
Ag	y = 0.000352x + 0.000206	0.950	0.5–5.0	15	3.3 × 10^−5^	4.8 × 10^−5^	0.15	0.45
Al	y = 0.0071x + 0.0538	0.995	39–2000	6	2.4 × 10^−5^	2.8 × 10^−2^	13	39
Br	y = 0.00949x + 0.0377	0.924	0.9–5.7	11	9.8 × 10^−4^	2.7 × 10^−3^	0.31	0.93
Ca	y = 0.000301x + 0.141	0.934	217–1740	12	1.7 × 10^−5^	2.0 × 10^−2^	72	217
Cd	y = 0.0098x + 0.141	0.794	0.07–0.12	4	5.5 × 10^−3^	2.6 × 10^−2^	0.02	0.07
Co	y = 0.0086x + 0.0014	0.864	0.3–0.6	4	3.1 × 10^−3^	3.4 × 10^−4^	0.10	0.30
Cu	y = 0.001813x + 0.00452	0.978	2.3–39.6	13	5.6 × 10^−6^	1.3 × 10^−3^	2.3	2.3
Cr	y = 0.00452x + 0.00779	0.913	0.7–8.8	15	2.6 × 10^−4^	9.9 × 10^−4^	0.24	0.72
Fe	y = 0.008972x + 0.0414	0.999	1.8–160.0	8	8.0 × 10^−5^	5.0 × 10^−3^	0.6	1.8
K	y = 0.000333x + 0.1199	0.969	24–510	10	1.4 × 10^−5^	2.5 × 10^−3^	8.4	25
Mg	y = 0.000053x + 0.0034	0.824	101–570	15	0.8 × 10^−5^	1.6 × 10^−3^	61	201
Mn	y = 0.00587x + 0.0036	0.969	1.3–6.0	9	8.1 × 10^−4^	2.2 × 10^−3^	0.4	1.3
Mo	y = 0.00091x + 0.000066	0.884	0.21–1.5	8	2.2 × 10^−4^	1.4 × 10^−5^	0.07	0.21
Na	y = 0.00091x + 0.000066	0.890	96–1599	5	7.6 × 10^−5^	5.2 × 10^−3^	137	417
Ni	y = 0.001135x + 0.00370	0.993	0.4–5.8	28	6.0 × 10^−5^	1.5 × 10^−4^	0.13	0.40
Pb	y = 0.002727x − 0.00035	0.984	0.4–6.8	10	8.3 × 10^−5^	3.1 × 10^−4^	0.13	0.38
S	y = 0.000035x − 0.263	0.983	7300–69,800	5	0.2 × 10^−5^	7.6 × 10^−2^	2431	7294
Se	y = 0.005864x − 0.00034	0.995	0.1–5.3	12	8.7 × 10^−5^	1.8 × 10^−4^	0.03	0.10
Si	y = 0.000069x − 0.0058	0.984	371–720	3	1.3 × 10^−5^	7.8 × 10^−3^	124	371
Sr	y = 0.000226x + 0.000325	0.970	1.1–9.2	7	1.1 × 10^−5^	7.6 × 10^−5^	0.37	1.11
V	y = 0.0072x + 0.016	0.999	4.5–43	12	1.0 × 10^−4^	2.8 × 10^−3^	1.5	4.5
Zn	y = 0.002505x + 0.035	0.974	16–251	13	8.4 × 10^−5^	1.2 × 10^−2^	5.3	16

LOD—limit of detection established using the equation: LOD = (3,3s)/a, where a—was the slope of the standard curve described by the equation y = ax + b and s was the standard deviation obtained for blank sample (n = 5), LOQ—limit of quantitation established as 3 × LOD.

**Table 2:** Comparison of instrumental versus preparative repeatability (pellet formation) for selected elements (* value below LOQ).

Element	Average Amount [µg g^−1^]	RSD for One Pellet (n = 3) [%]	RSD for Three Pellets [%]
S	49,423	0.26	3.6
Ca	1190	0.63	4.4
Ag	0.217 *	9.4	9.8
Fe	25.9	0.33	5.0
Cu	11.04	1.6	2.6
Si	531	3.9	5.5
Zn	100.8	0.34	4.9
Se	0.587	2.3	4.3
Sr	6.190	2.6	2.8
Pb	4.116	1.1	1.6

**Table 3:** Comparison of elements’ amounts established by EDX with certified amounts for analyzed reference material made of hair (DC/NC—the type of reference material with indicated (○-dis and, ●-agreement). Recovery (RE) and coefficient of variation (CV) are presented for the EDX method.

Element	C_DC-CRM_ ± U	C_DC-EDX_ ± U	RE_DC_ [%]	CV_DC_ [%]	DC/NC Agreed
Ag	0.050 ± 0.005	<LOD	N/A	N/A	○/○
Al	20,000 ± N/A	19,710 ± 190	99	1.9	●/●
Br	1.10 ± N/A	0.989 ± 0.014	90	2.9	●/○
Ca	1450 ± 200	1326 ± 35	91	5.3	●/●
Cd	0.070 ± 0.010	0.0857 ± 0.0031	122	7.2	●/○
Cl	180 ± 21	163.9 ± 3.7	91	4.5	●/○
Co	0.045 ± 0.009	<LOD	N/A	N/A	○/●
Cr	0.41 ± 0.12	0.329 ± 0.079	80	4.8	●/●
Cu	14.3 ± 1.6	16.2 ± 1.9	113	2.4	●/●
Fe	36.0 ± 5.0	35.10 ± 0.93	98	5.3	●/●
K	20.0 ± 1.8	17.93 ± 0.20	90	2.2	●/○
Mg	140 ± 14	134.5 ± 6.9	96	10	●/●
Mn	2.0 ± 0.3	1.84 ± 0.25	92	13	●/●
Mo	0.17 ± 0.03	<LOD	N/A	N/A	○/●
Na	89 ± 12	<LOD	N/A	N/A	○/●
Ni	0.43 ± 0.12	0.53 ± 0.14	123	26	●/●
Pb	5.7 ± 0.5	5.58 ± 0.22	98	7.9	●/●
S	41,900 ± 1100	41,760 ± 293	100	1.4	●/●
Se	0.58 ± 0.12	0.600 ± 0.036	103	12	●/●
Si	600 ± N/A	613 ± 14	102	4.5	●/●
Sr	7.70 ± 0.40	7.81 ± 0.14	101	3.6	●/●
V	0.50 ± 0.18	<LOD	N/A	N/A	○/○
Zn	137 ± 9	138.8 ± 1.9	101	2.7	●/●

**Table 4:** Comparison of amounts of elements (μg g^−1^) established by ICP-MS/MS and EDX nail pulled sample. Uncertainty of the method was calculated for k = 2. Values are in agreement when |C_ICP-MS_ − C_EDX_| ≤ ∑U.

Element	C_ICP-MS_ ± U	C_EDX_ ± U	|C_ICP-MS_ − C_EDX_|	∑U	Agreement
Ag	1.45 ± 0.94	2.52 ± 0.98	0.07	0.14	√
Al	29.2 ± 5.9	N/A	N/A	N/A	N/A
Ca	1596 ± 359	1891 ± 267	295	447	√
Cd	0.022 ± 0.009	0.011 ± 0.005	0.012	0.011	-
Cr	2.18 ± 0.79	2.34 ± 0.38	0.16	0.88	√
Cu	4.07 ± 1.37	3.77 ± 0.68	0.30	1.53	√
Fe	15.3 ± 3.7	10.8 ± 3.0	4.5	4.8	√
K	154 ± 39	133 ± 81	21	21	√
Mg	246 ± 68	224 ± 55	42	87	√
Mn	5.08 ± 0.30	4.84 ± 0.25	0.24	0.39	√
Mo	2.98 ± 0.58	N/A	N/A	N/A	N/A
Na	139 ± 62	N/A	N/A	N/A	N/A
Ni	3.89 ± 0.22	3.63 ± 0.32	0.26	0.38	√
Pb	0.26 ± 0.11	0.21 ± 0.17	0.06	0.21	√
S	33,247 ± 1829	31,638 ± 1265	1609	2223	√
Se	0.51 ± 0.14	0.44 ± 0.10	0.07	0.17	√
Si	306 ± 88	263 ± 34	43	94	√
Sr	3.33 ± 0.98	3.11 ± 0.91	0.22	1.3	√
Zn	125 ± 50	111 ± 18	14	53	√

N/A—not available.

**Table 5:** Composition of calibration pellets for EDX.

	Method A *: CRM Diluted with Binder	Method B: CRM Fortified with ECS
Used CRMs	NCS DC73347a/NCS ZC 8100 2b/ERM-DB001	NCS DC73347a
Amount of CRM [mg]—x	120, 100, 80, 60	50
Volume of ECS solution [µL]	N/A	5, 10, 20, 50
Volume of TMAHMeOH [µL]	250	250
VMeOH [µL]	50	50
Amount of boric acid [mg]	250	250

x—weight of CRM according to Equation (1) (mg); * method validated and recommended.

**Table 6:** Optimal conditions for metal determination by EDX and ICP MS/MS. See Table S3 for more detailed information.

Parameter	Value
X-ray fluorescence spectrometry	
Instrument model and mode	Model EDX-8100, Vacuum (<5 Pa)
X-ray lamp	Rh
Collimator	10 mm
Generator current XRF	Medium: 100 μA (automatic, filter dependent)
Generator voltage XRF, Scanning time, Dead Time (DT) for two groups of elements	15 kV, 400 s, DT 30%—Al, Ca, K, Na, Mg, P, S 50 kV, 600 s, DT 30%—As, Ag, Cd, Fe, Mn Se, Zn, Cu, Pb, Br, Sr, Y, Tl, U
Filters assigned for the determination of appropriate elements	No filter—Al *, Na *, Mg *, P *, Si * 1—Ag *, Cd *, Mo *, Rb *, Sb *, Sr *, Y *, Tl ***, U *** 2—Ba ***, Ca *, Cl *, K *, S *, Ti *, V * 3—Cr *, Fe *, Mn * 4—As **, Br *, Co *, Cu *, Hg ***, Ni *, Pb ***, Se *, Zn *,
Inductively coupled plasma mass spectrometry, ICP-MS/MS	
Model	8900, QqQ
Generator RF power	1550 W
Nebulizer gas flow	1.05 L∙min^−1^
Collision gas flow	[No gas], [He] 5.5 mL∙min^−1^, [O2] 0.45 mL∙min^−1^
Gas flow for high matrix dilution	0.9 L∙min^−1^
Sampling program	Sample injection: 50 s, stabilization: 40 s, acquisition: 30 s
Integration times for monitored isotopes	5 ms: ^56^Fe, 10 ms: ^24^Mg, ^27^Al 50 ms: ^31^P, ^39^K, ^44^Ca, ^45^Sc 100 ms: ^7^Li, ^9^Be, ^47^Ti, ^51^V, ^52^Cr, ^55^Mn, ^59^Co, ^60^Ni, ^63^Cu, ^66^Zn, ^71^Ga, ^72^Ge_IS_, ^88^Sr, ^89^Y_IS_, ^95^Mo, ^103^Rh_IS_, ^107^Ag, ^115^In, ^118^Sn, ^121^Sb, ^125^Te, ^127^I, ^137^Ba, ^201^Hg, ^205^Tl, ^206^Pb, ^209^Bi, ^232^Th, ^238^U 300 ms: ^75^As, ^78^Se, ^79^Br, ^23^Na 1000 ms: ^111^Cd

*, **, ***—signals selected to monitor elements related to X-ray emitted when replenishing the K and L shells with electron—K_α_, K_β_, L_α_, respectively.

## Data Availability

The data used to support the findings of this study are available from the corresponding author upon request.

## Supplementary Materials

The following supporting information can be downloaded at: https://www.mdpi.com/article/10.3390/molecules29040773/s1, Figure S1: The comparison of the homogeneity of milled hair offered by the European Research Institute (JRC) (a), the Chinese Research Institute (b), and those produced within the project (c), Figure S2: Effect of pellet thickness on changes in signal intensity and relative standard deviation (RSD); Figure S3: Comparison of amounts for selected elements in CRM of human hair established by EDX and ICP MS/MS; Table S1: Mineralization program for hair and nail samples toward ICP MS/MS analysis, Table S2: The volumes of reagents used for the alkaline extraction of metals from a sample of hair and nails toward ICP MS/MS analysis, Table S3: Statistical description of calibration curves obtained by ICP MS/MS in 2% HNO_3_, Table S4: Statistical description of calibration curves obtained by ICP MS/MS in 1% TMAH, Table S5: Comparison of the metals’ amounts established by ICP MS/MS with certified values obtained for CRM (NCS ZC 81OO2b human hair). (unit μg/g), Table S6: Statistical description of calibration curves obtained by EDX using different methods for pellets’ preparation (unit μg/g), Section S3: Modification of PCEDX-Pro Ver.2.05 software.
