# Supplementary material for: Multi-Elemental Analysis of Hair and Fingernails Using Energy-Dispersive X-ray Fluorescence (ED XRF) Method Supported by Inductively Coupled Plasma Mass Spectrometry (ICP MS)
Source: Molecules. 2024 Feb 7;29(4):773. doi: 10.3390/molecules29040773 (PMC10892256; doi:10.3390/molecules29040773)
Supplement: Supplementary file 1 [file molecules-29-00773-s001.zip › molecules-2824974-supplementary.pdf]

Supporting Information

for:

Multi-Elemental Analysis of Hair and Fingernails Using Energy-Dispersive X-ray Fluorescence (ED XRF) Method Supported by Inductively Coupled Plasma Mass Spectrometry (ICP MS)

Section S1. Tables

Table S1. Mineralization program for hair and nail samples toward ICP MS/MS analysis

TableS2. The volumes of reagents used for the alkaline extraction of metals from a sample of hair and nails toward ICP-MS/MS analysis

Table S3. Statistical description of calibration curves obtained by ICP MS/MS in 2% HNO_3

Table S4. Statistical description of calibration curves obtained by ICP MS/MS in 1% TMAH

Table S5. Comparison of the metals' amounts established by ICP MS/MS with certified values obtained for CRM (NCS ZC 81OO2b human hair). (unit μg/g)

Table S6. Statistical description of calibration curves obtained by EDX using different methods for pellets' preparation (unit μg/g)

Section S2. Figures

Figure S1. The comparison of the homogeneity of milled hair offered by the European Research Institute (JRC) (a), the Chinese Research Institute (b), and those produced within the project (c)

Figure S2. Effect of pellet thickness on changes in signal intensity and relative standard deviation (RSD)

Figure S3. Comparison of amounts for selected elements in CRM of human hair established by EDX and ICP MS/MS

Section S3. Modification of PCEDX-Pro Ver.2.05 software

Section 1. Tables

Table S1. Mineralization program for hair and nail samples toward ICP MS/MS analysis

Step	1	2	3	4	5
T [°C]	160	190	50	50	50
P [bar]	40	40	0	0	0
Power [%]	80	90	0	0	0
Time [min]	10	15	5	1	1

TableS2. The volumes of reagents used for the alkaline extraction of metals from a sample of hair and nails toward ICP MS/MS analysis

**Solution added (concentration)**	**hair**	**nails**
**TMAH (25%, m/v)**	5 mL	2.5 mL
**Thiourea (1%, m/v)**	0.5 mL	0.25 ml
**Triton (1%, m/v)**	1 mL	0.5 ml
**EDTA (10%, m/v)**	0.5 mL	0.25 ml
**MQ-water**	13 mL	6.5 mL

Table S3. Statistical description of calibration curves obtained by ICP MS/MS in 2% HNO_3

Isotope	r^2	a	b	SD_a	SD_b	IDL* [ppb]	MDL* [ppb]	LOQ* [ppb]
^{23}Na [He]	0.998	0.090	2.199	0.002	3.950	21.321	144.5	433.6
^{24}Mg [He]	0.999	0.090	1.608	0.002	2.662	4.720	97.2	291.6
^{27}Al [He]	0.966	0.094	0.081	0.008	0.141	10.323	4.98	14.94
$^{31\text{->}47}$P [O_2]	1.000	0.089	0.003	0.001	0.012	0.205	0.44	1.32
$^{32\text{->}48}$S [O_2]	1.000	0.089	0.025	0.002	2.251	12.542	33.72	89.8
$^{35\text{->}35}$Cl [O_2]	0.995	0.090	20.02	0.001	9.021	56.456	86.76	172.28
^{39}K [He]	0.999	0.090	2.076	0.002	3.162	5.878	115.7	346.9
^{44}Ca [He]	0.999	0.090	3.406	0.002	3.418	25.954	125.7	377
$^{51\text{->}67}$V [O_2]	1.000	0.089	0.001	0.001	0.013	0.001	0.47	1.39
$^{52\text{->}68}$Cr [O_2]	1.000	0.089	0.009	0.001	0.014	0.017	0.53	1.58
$^{55\text{->}71}$Mn [O_2]	1.000	0.089	0.010	0.001	0.011	0.009	0.41	1.24
^{56}Fe [He]	1.,000	0.091	0.784	0.001	1.474	0.603	53.6	160.9
^{59}Co [He]	1.000	0.091	0.011	0.001	0.018	0.004	0.65	1.94
^{60}Ni [He]	1.000	0.090	0.013	0.001	0.017	0.025	0.63	1.90
^{63}Cu [He]	1.000	0.090	0.015	0.001	0.023	0.055	0.83	2.48
^{66}Zn [He]	1.000	0.091	0.004	0.001	0.011	0.117	0.39	1.18
^{75}As [He]	1.000	0.089	0.020	0.001	0.020	0.026	0.73	2.18
$^{75\text{->}91}$As [O_2]	1.000	0.090	0.000	0.001	0.010	0.014	0.38	1.13
^{78}Se [He]	1.000	0.090	0.003	0.001	0.012	0.114	0.44	1.33
$^{78\text{->}94}$Se [O_2]	1.000	0.090	-0.004	0.001	0.010	0.046	0.35	1.05
^{79}Br [He]	0.999	0.089	0.013	0.001	0.019	0.859	0.7	2.11
^{88}Sr [He]	1.000	0.090	0.010	0.001	0.017	0.236	0.61	1.84
^{95}Mo [He]	1.000	0.090	0.011	0.001	0.017	0.002	0.62	1.86
^{107}Ag [He]	0.993	0.073	-0.085	0.005	0.087	0.034	3.90	11.7
^{111}Cd [He]	1.000	0.092	-0.008	0.000	0.008	0.002	0.27	0.81
^{118}Sn [He]	1.000	0.094	-0.044	0.001	0.014	0.043	0.51	1.53
$^{121\text{->}137}$Sb [O_2]	1.000	0.092	-0.009	0.000	0.005	0.013	0.19	0.55
^{127}I [No Gas]	0.988	0.078	0.084	0.004	0.062	0.063	2.63	7.88
^{138}Ba [He]	0.999	0.092	0.016	0.001	0.024	0.053	0.85	2.54
^{201}Hg [He]	0.996	0.093	-0.042	0.005	0.087	0.064	3.08	5.23
^{205}Tl [He]	1.000	0.092	-0.003	0.001	0.010	0.001	0.34	1.03
^{207}Pb [He]	1.000	0.092	-0.006	0.000	0.006	0.029	0.21	0.64
^{209}Bi [He]	0.993	0.091	0.154	0.012	0.207	2.674	7.50	22.5
^{232}Th [He]	1.000	0.093	-0.020	0.001	0.009	0.001	0.32	0.97
^{238}U [He]	1.000	0.093	-0.012	0.000	0.008	0.001	0.28	0.83

Table S4. Statistical description of calibration curves obtained by ICP MS/MS in 1% TMAH

**Isotope**	**r^2**	**a**	**b**	**SD_a**	**SD_b**	**IDL* [ppb]**	**MDL* [ppb]**	**LOQ* [ppb]**
**^{23}Na [He]**	0.998	0.021	0.058	0.001	6.527	51	219	653
**^{24}Mg [He]**	1.000	0.018	-0.182	$1.7\cdot10^{-4}$	0.292	1.051	53.7	161
**^{27}Al [He]**	0.991	0.005	0.043	$2.3\cdot10^{-4}$	0.004	2.966	2.45	7.36
**$^{31->47}$P [O_2]**	0.997	0.300	8.597	0.008	0.132	0.707	1.45	4.36
**$^{32->48}$S [O_2]**	0.998	0.320	21.326	0.009	10.254	15.2	45.2	141
**$^{35->35}$Cl [O_2]**	0.998	0.033	45.002	$2.2\cdot10^{-4}$	13.004	22.0	40.9	121.7
**^{39}K [He]**	1.000	0.015	0.302	$1.3\cdot10^{-4}$	0.223	2.100	49.3	148
**^{44}Ca [He]**	0.998	0.001	0.219	$1.4\cdot10^{-5}$	0.025	23.44	103.7	311
**$^{51->67}$V [O_2]**	1.000	11.901	-1.903	0.100	1.746	0.004	0.48	1.45
**$^{52->68}$Cr [O_2]**	1.000	0.475	0.062	0.001	0.019	0.026	0.13	0.40
**$^{55->71}$Mn [O_2]**	1.000	0.284	0.040	0.001	0.025	0.035	0.29	0.87
**^{56}Fe [He]**	0.999	0.292	-3.080	0.005	8.324	7.724	93.9	282
**^{59}Co [He]**	0.999	0.684	0.045	0.007	0.125	0.000	0.6	1.81
**^{59}Co [He]**	1.000	0.191	0.038	0.002	0.031	0.036	0.53	1.60
**^{63}Cu [He]**	0.999	0.535	0.195	0.006	0.107	0.022	0.66	1.98
**^{66}Zn [He]**	0.996	0.068	0.348	0.002	0.034	0.39	1.67	5.00
**^{75}As [He]**	0.999	0.067	0.010	0.001	0.014	0.019	0.69	2.06
**$^{75->91}$As [O_2]**	1.000	0.016	0.001	$5.8\cdot10^{-5}$	0.001	0.000	0.21	0.63
**^{78}Se [He]**	1.000	0.005	-0.001	$4.5\cdot10^{-5}$	$4.2\cdot10^{-4}$	0.074	0.31	0.92
**$^{78->94}$Se [O_2]**	1.000	$3.3\cdot10^{-4}$	$1.1\cdot10^{-5}$	$7.5\cdot10^{-7}$	$7.0\cdot10^{-6}$	0.030	0.070	0.21
**^{79}Br [He]**	0.998	0.001	0.001	$1.4\cdot10^{-5}$	$2.5\cdot10^{-4}$	0.741	1.284	3.85
**^{88}Sr [He]**	1.000	0.08	0.036	$3.4\cdot10^{-4}$	0.006	0.016	0.240	0.72
**^{95}Mo [He]**	1.000	0.058	0.002	$2.7\cdot10^{-4}$	0.005	0.018	0.271	0.81
**^{107}Ag [He]**	1.000	0.055	0.007	0.001	0.009	0.009	0.556	1.67
**^{111}Cd [He]**	1.000	0.005	$-1.4\cdot10^{-5}$	$1.6\cdot10^{-5}$	$2.8\cdot10^{-4}$	0.007	0.187	0.56
**^{118}Sn [He]**	1.000	0.01	0.001	$3.3\cdot10^{-5}$	0.001	0.009	0.196	0.59
**$^{121>137}$Sb [O_2]**	1.000	0.852	-0.016	0.006	0.097	0.003	0.376	1,13
**^{127}I [No Gas]**	0.999	0.003	0.002	$3.7\cdot10^{-5}$	0.001	0.128	0.623	1.87
**^{138}Ba [He]**	0.998	0.025	0.059	$4.9\cdot10^{-4}$	0.009	0.094	1.162	3.49
**^{201}Hg [He]**	0.999	0.006	0.002	$8.6\cdot10^{-5}$	0.001	0.132	0.795	2.38
**^{205}Tl [He]**	1.000	0.078	0.005	$4.9\cdot10^{-4}$	0.009	0.001	0.363	1.09
**^{207}[Pb] [He]**	1.000	0.022	0.007	$1.7\ 10^{-4}$	0.003	0.023	0.455	1.37
**^{209}Bi [He]**	0.996	$4.3\cdot10^{-5}$	$2.3\cdot10^{-4}$	$1.2\cdot10^{-6}$	$2.1\cdot10^{-5}$	0.501	1.601	4.80
**^{232}Th [He]**	1.000	0.104	-0.006	$4.5\cdot10^{-4}$	0.008	0.001	0.246	0.74
**^{238}U [He]**	1.000	0.113	-0.004	$3.9\cdot10^{-4}$	0.007	0.001	0.200	0.60

*LOD was established using equation:

$$LOD = \frac{3{,}3s}{a}, \text{ where}$$

a - was the slope of the standard curve described by the equation y = ax + b

s – standard deviation of the standard curve $s_{y/x}$ – LOD assigned as IDL

s – standard deviation of b s_b – LOD assaigned as MDL

The limit of quantification was calculated as: LOQ = MDL*3

Table S5. Comparison of the metals' amounts established by ICP MS/MS with certified values obtained for CRM (NCS ZC 81OO2b human hair). (unit µg/g)

Element	C_{CRM} ± U	C_{determ} ±U	RE [%]	CV [%]	Egreement
Na	445 ± 40	460 ± 14	3.0	5.3	+
Mg	248 ± 14	202 ± 29	1.5	22.1	+
Al	23.2 ± 2.0	26.1 ± 2.4	33.6	9.5	+
K	14.4 ± 1.4	14.50 ± 0.45	9.7	0.1	+
Ca	1537 ± 68	1525 ± 195	12.8	2.1	+
V	0.089 ± 0.009	0.09 ± 0.01	6.7	5.7	+
Cr	8.74 ± 0.97	8.69 ± 1.11	0.3	5.2	+
Mn	3.83 ± 0.39	3.82 ± 0.24	8.3	4.1	+
Fe	160 ± 16	158 ± 20	6.8	2.0	+
Co	0.153 ± 0.015	<LOD	2.8	N/A	N/A
Ni	5.77 ± 0.577	5.66 ± 0.69	3.7	3.2	+
Cu	33.6 ± 2.3	34.1 ± 4.9	1.2	3.8	+
Zn	191 ± 16	186 ± 20	12.4	0.7	+
Se	0.59 ± 0.04	0.58 ± 0.10	55.4	19.6	+
Sr	8.17 ± 0.69	7.75 ± 3.99	9.3	2.7	+
Mo	1.06 ± 0.12	1.00 ± 0.12	6.7	2.6	+
Cd	0.07 ± 0.01	0.08 ± 0.01	11.7	9.9	+
Ba	11.1 ± 1.3	11.9 ± 1.53	37.6	6.0	+
Pb	3.83 ± 0.18	3.9 ± 0.5	3.6	2.7	+

N/A – not available

Table S6. Statistical description of calibration curves obtained by EDX using different methods for pellets' preparation (unit μg/g)

	Pellets made of grinded hair with known amounts of metals (CRM/LRM), (G)			Standard solutions of metal ions with gelatin dissolved in TMAH-H_2O (TH)			Hair with known amounts of metals (CRM/LRM) dissolved in TMAH-MeOH (TM)and diluted with different amount of boric acid		
El.	y = ax + b	r^2	LOD	y = ax + b	r^2	LOD	y = ax + b	r^2	LOD
Ag	Not detected	N/A	N/A	y = 0.000229x + 0.000179	0.960	1.4	y = 0.000352x + 0.000206	0.950	0.15
Br	y = 0.019928x + 0.001904	0.994	0.06	N/A	N/A	N/A	y = 0.009486x + 0.037724	0.924	0.3
Ca	y = 0.001280x – 0.002171	0.850	0.45	y = 0.005147x + 0.136258	0.999	7.1	y = 0.000301x + 0.141035	0.934	27.6
Cl	y = 0.000208x + 0.001556	0.866	11.5	N/A	N/A	N/A	y = 0.000102x + 0.001245	0.921	5.4
Cr	y = 0.012631x + 0.009551	0.966	1.33	y = 0.018969x + 0.007320	0.996	0.44	y = 0.004518x + 0.007795	0.913	0.24
Cu	y = 0.005573x – 0.005698	0.979	0.24	y = 0.001902x + 0.003864	0.987	0.41	y = 0.001813x + 0.004518	0.978	0.38
Fe	y = 0.026508x + 0.057074	0.999	0.71	y = 0.026506x + 0.110466	0.999	0.55	y = 0.008972x + 0.041425	0.999	0.64
Hg	y = 0.007095x – 0.003147	0.943	0.08	N/A	N/A	N/A	y = 0.002365x – 0.002518	0.921	0.21
K	Not detected	N/A	N/A	y = 0.000611x + 0.005719	0.999	6.4	y = 0.000333x + 0.119939	0.969	7.7
Mg	y = 0.000220x – 0.003265	0.757	7.7	y = 0.002062x + 0.043004	0.986	23.7	y = 0.000053x + 0.003351	0.824	31
Mn	y = 0.018890x – 0.000052	0.986	0.51	y = 0.018811x + 0.002758	0.998	0.36	y = 0.005868x + 0.003563	0.969	0.4
Na	y = 0.000188x – 0.004879	0.946	38.1	N/A	N/A	N/A	y = 0.000047x – 0.006279	0.924	60
Ni	y = 0.003521x + 0.003058	0.995	0.22	y = 0.001584x + 0.002088	0.991	0.45	y = 0.001135x + 0.003703	0.993	0.12
Pb	y = 0.007734x – 0.001161	0.999	0.21	y = 0.007737x + 0.000289	0.999	0.09	y = 0.002727x – 0.000354	0.984	0.14
S	y = 0.000142x + 0.162109	0.978	245	N/A	N/A	N/A	y = 0.000035x – 0.263068	0.983	30.7

Se	y = 0.013036x + 0.003004	0.996	0.09	y = 0.004447x + 0.000473	0.995	0.11	y = 0.005864x – 0.000337	0.995	0.06
Si	Not detected	N/A	N/A	N/A	N/A	N/A	y = 0.000069x – 0.005833	0.984	0.14
Sr	y = 0.028404x – 0.059188	0.694	0.15	N/A	N/A	N/A	y = 0.000226x + 0.000325	0.970	0.07
Zn	y = 0.007682x – 0.062944	0.976	0.12	y = 0.004034x + 0.008687	0.627	3.1	y = 0.002505x + 0.035091	0.974	0.40

N/A – not available. Prepared standard mixture with gelatin was not homogeneous and the linear range for calibration graph was not achievable with EDX; LOD –the limit of detection was determined based on the standard deviation of the blank sample (TH)and for deionized hair (calibration curve G and TM)
G – calibration curves obtained via method A, TH, TM - calibration curves obtained via method B (Table 5)

Section 2. Figures

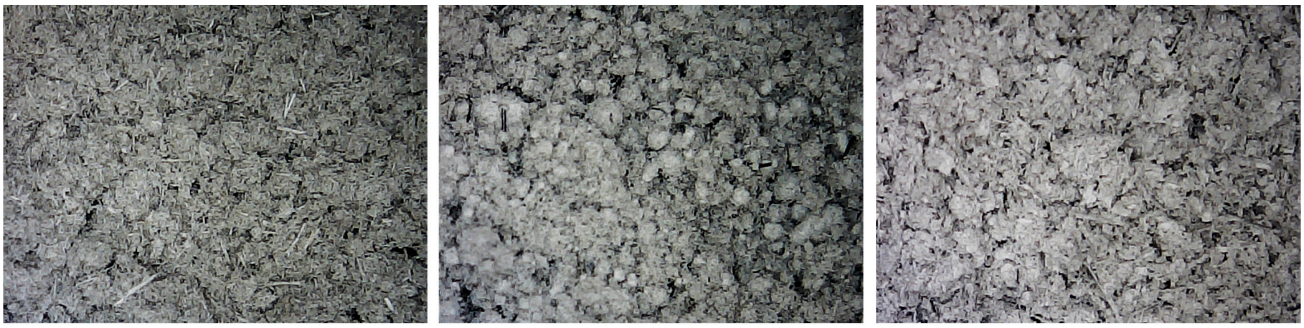

(a) Hair ERM – DB001 (b) Hair NCS ZC 81002b (c) Hair grinded within the project

Figure S1. The comparison of the homogeneity of milled hair offered by the European Research Institute (JRC) (a), the Chinese Research Institute (b), and those produced within the project (c).

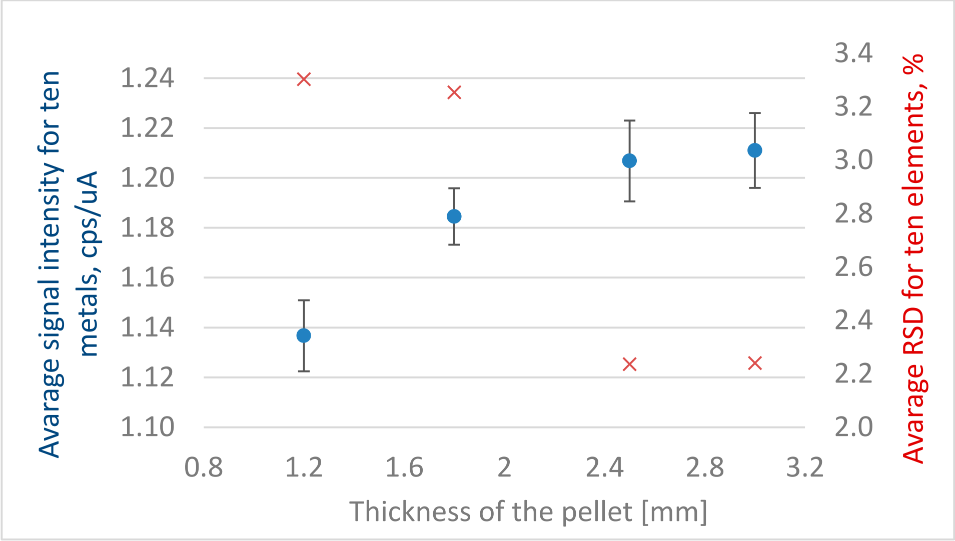

Figure S2. Effect of pellet thickness on changes in signal intensity and relative standard deviation (RSD).

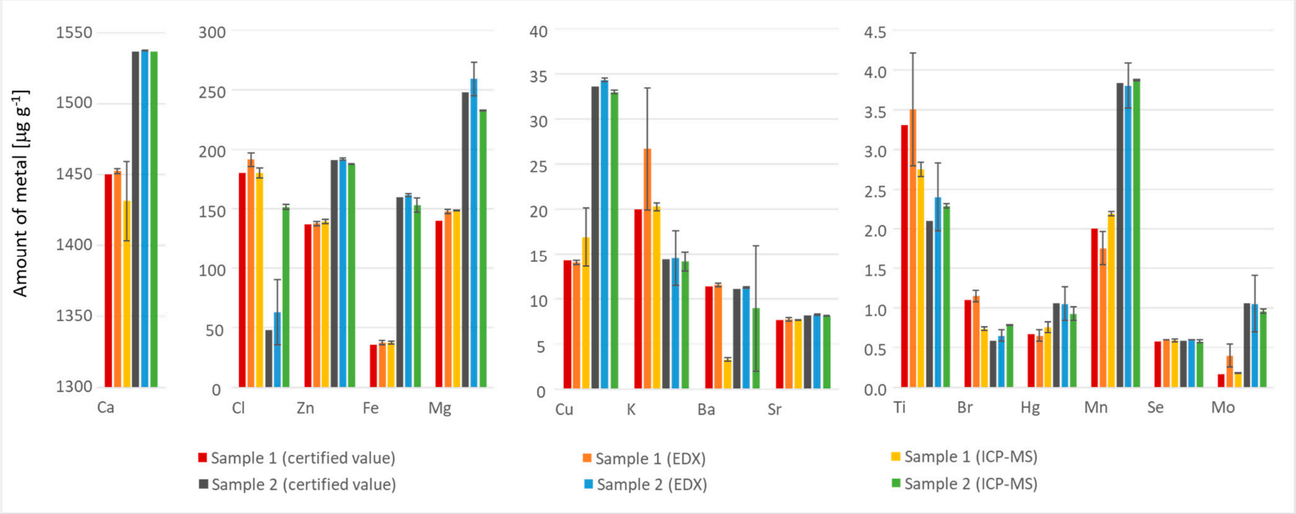

Figure S3. Comparison of amounts for selected elements in CRM of human hair established by EDX and ICP MS/MS

Section 3. Modification of PCEDX-Pro Ver.2.05 software

Errors were detected in macros used by the PCEDX-Navi software: one macro, when creating a report, removed the last four decimal digits, leaving only the first two of six, using critical parameters described in the file saving camera calibration data (InsertDispMeasChanToPage, InsertEDXContents module); the second macro downloaded the spectrum obtained for the first measurement conditions without taking into account hybrid methods (MakeGroup, GraphContents module and edxtext4e.exe program), in which different energy ranges are used using different filters. The report was proofread by trial and error and by detecting errors in report templates (BasicReportV004a-en) because comments in the software were written in Japanese, some were obsoleted, and parts of software code were obfuscated using different methods, making it challenging to identify the functions of individual script sections.

In order to extract more accurate data from the PCEDX program, the possibility of external access to data, direct access to result files, and interception of data shared by individual components of the application was first checked. Based on the provided data, a template and scripts were prepared, which made it possible to generate a report with an accuracy of up to 4 decimal places; obtaining higher accuracy was impossible because the data placed by the program in the temporary files sr_text.txt after data registration is only accurate to 4 decimal places. It was not possible to modify the tool at this level.

Additionally, a new tool was created in the PCEDX-Navi software to determine the variance and standard deviation of the test result provided in the report based on the averaged result from any number of scans. The original software determined the standard deviation for the obtained results, which did not correctly reflect the method variance. The most straightforward indicator was the lack of correlation between the increase in the standard deviation and the decrease in the determined content. This relationship is evident because as the analyte concentration decreases, the signal height (S) decreases relative to the constant noise level (N). We hypothesized that the software determines the signal height's standard deviation based on the theoretical curve's fit to the experimental spectrum curve. After performing a series of calculations and examining the degree of fit of the spectrum to the mathematically described curve in the Fityk program, we noticed that the determined standard deviations were similar to those given in the report generated by the PCEDX-Navi software. Therefore, the software was modified to determine the variance (from which the standard deviation can be determined) based on changes in the signal heights obtained for the analyte for repeated scans.

Since changing the PCDEX-Navi program was impossible, an alternative approach was used - capturing data directly displayed in PCEDX in the standard sample form, where it occurs with the required accuracy. Due to the structure of the program, standard data export was difficult, so it was decided to prepare an automatic tool based on the so-called screen scraping (retrieving information from one application window and transferring it to another application window) in order to download the necessary data from PCEDX, prepare it for the appropriate format and export it to .xls files. Several available tools were tested, and during the tests, there appeared to be problems with the non-standard behavior of the PCEDX application (anomalies occurring with randomly opening windows in non-standard places, including outside the screen area, covering windows, and with delay that was difficult to determine). The best performance was obtained using the tool available under the SikuliX open license. Data extraction operations were automated using SikuliX, but due to the low accuracy of OCRs (Optical Character Recognition software), both those associated with SikuliX and other tested programs causing unacceptable errors (changing a digit to a similar one, omitting one digit) in 2-3% of cases, it was decided to add an additional pre-processing step related to exporting to PDF and then processing data in text format in order to prepare them for import into a spreadsheet.

After modifying the macros and developing a data transfer tool (using SikuliX 2.0.1), the tool was installed on the computer to record the spectra. The test stage was carried out, which consisted of

exporting the data obtained for 30 different samples to a single spreadsheet, for which the compliance of the measurement data with the imported data in the spreadsheet was checked. It was also confirmed that the changing number of elements in the report and changes in the parameters of the measurement method do not affect the effectiveness of the modified macros.
